# Determinants of Atherogenic Dyslipidemia and Lipid Ratios: Associations with Sociodemographic Profile, Lifestyle, and Social Isolation in Spanish Workers

**DOI:** 10.3390/jcm14197039

**Published:** 2025-10-05

**Authors:** Pere Riutord-Sbert, Pedro Juan Tárraga López, Ángel Arturo López-González, Irene Coll Campayo, Carla Busquets-Cortés, José Ignacio Ramírez Manent

**Affiliations:** 1 ADEMA-Health Group, University Institute for Research in Health Sciences (IUNICS), 07122 Palma, Spain; 2 Faculty of Medicine, University of Castilla La Mancha (UCLM), 02008 Albacete, Spain; pjtarraga@sescam.jccm.es; 3 SESCAM (Health Service of Castilla La Mancha), 02008 Albacete, Spain; 4 Balearic Islands Health Research Institute Foundation (IDISBA), 07010 Palma, Spain; 5 Balearic Islands Health Service, 07010 Palma, Spain; 6 Faculty of Medicine, University of the Balearic Islands, 07010 Palma, Spain

**Keywords:** atherogenic dyslipidemia, atherogenic indices, sociodemographic variables, lifestyle, Mediterranean diet, social isolation

## Abstract

**Background:** Atherogenic dyslipidemia is defined by the coexistence of high triglyceride concentrations, low levels of high-density lipoprotein cholesterol (HDL-C), and an excess of small, dense particles of low-density lipoprotein cholesterol (LDL-C). This lipid profile is strongly associated with an increased burden of cardiovascular disease and represents a leading cause of global morbidity and mortality. To better capture this risk, composite lipid ratios—including total cholesterol to HDL-C (TC/HDL-C), LDL-C to HDL-C (LDL-C/HDL-C), triglycerides to HDL-C (TG/HDL-C), and the atherogenic dyslipidemia index (AD)—have emerged as robust markers of cardiometabolic health, frequently demonstrating superior predictive capacity compared with isolated lipid measures. Despite extensive evidence linking these ratios to cardiovascular disease, few large-scale studies have examined their association with sociodemographic characteristics, lifestyle behaviors, and social isolation in working populations. **Methods:** We conducted a cross-sectional analysis of a large occupational cohort of Spanish workers evaluated between January 2021 and December 2024. Anthropometric, biochemical, and sociodemographic data were collected through standardized clinical protocols. Indices of atherogenic risk—namely the ratios TC/HDL-C, LDL-C/HDL-C, TG/HDL-C, and the atherogenic dyslipidemia index (AD)—were derived from fasting lipid measurements. The assessment of lifestyle factors included tobacco use, physical activity evaluated through the International Physical Activity Questionnaire (IPAQ), adherence to the Mediterranean dietary pattern using the MEDAS questionnaire, and perceived social isolation measured by the Lubben Social Network Scale. Socioeconomic classification was established following the criteria proposed by the Spanish Society of Epidemiology. Logistic regression models were fitted to identify factors independently associated with moderate-to-high risk for each lipid indicator, adjusting for potential confounders. **Results:** A total of 117,298 workers (71,384 men and 45,914 women) were included. Men showed significantly higher odds of elevated TG/HDL-C (OR 4.22, 95% CI 3.70–4.75) and AD (OR 2.95, 95% CI 2.70–3.21) compared with women, whereas LDL-C/HDL-C ratios were lower (OR 0.86, 95% CI 0.83–0.89). Advancing age was positively associated with all lipid ratios, with the highest risk observed in participants aged 60–69 years. Lower social class, smoking, physical inactivity, poor adherence to the Mediterranean diet, and low social isolation scores were consistently linked to higher atherogenic risk. Physical inactivity showed the strongest associations across all indicators, with ORs ranging from 3.54 for TC/HDL-C to 7.12 for AD. **Conclusions:** Atherogenic dyslipidemia and elevated lipid ratios are strongly associated with male sex, older age, lower socioeconomic status, unhealthy lifestyle behaviors, and reduced social integration among Spanish workers. These findings highlight the importance of workplace-based cardiovascular risk screening and targeted prevention strategies, particularly in high-risk subgroups. Interventions to promote physical activity, healthy dietary patterns, and social connectedness may contribute to lowering atherogenic risk in occupational settings.

## 1. Introduction

Atherogenic dyslipidemia (AD) is a distinct lipid disorder characterized by elevated triglycerides and reduced high-density lipoprotein cholesterol (HDL-c) [1]. This lipid triad, which reflects a highly atherogenic profile, is strongly associated with an increased risk of cardiovascular disease (CVD), and is frequently observed in individuals with metabolic syndrome, abdominal obesity, type 2 diabetes mellitus, and insulin resistance [2,3,4,5]. However, AD is not restricted to these metabolic states; it can also be present in individuals without overt metabolic alterations, highlighting its clinical and epidemiological importance [6].

Evaluating atherogenic risk by means of lipid ratios—such as total cholesterol to HDL-C (TC/HDL-C), LDL-C to HDL-C (LDL-C/HDL-C), and triglycerides to HDL-C (TG/HDL-C)—has gained recognition as a practical, inexpensive, and dependable method for stratifying cardiovascular risk [7]. These ratios, derived from routine biochemical measurements, have demonstrated stronger predictive value for atherosclerotic events than isolated lipid concentrations [8]. Elevated values indicate an unfavorable balance between atherogenic and anti-atherogenic lipoproteins, facilitating the early identification of individuals at higher risk of coronary heart disease and cerebrovascular events [9,10].

The prevalence and distribution of AD and elevated lipid ratios vary substantially across populations, influenced by sociodemographic, behavioral, and environmental factors [11,12]. Evidence suggests that men tend to exhibit higher atherogenic lipid ratios than women, particularly during midlife, whereas postmenopausal women experience a deterioration in lipid profiles due to hormonal changes [13]. Aging is associated with progressive increases in TC/HDL-c and LDL-c/HDL-c ratios, along with a higher prevalence of AD [14]. Socioeconomic position also plays a key role, as individuals from lower social classes often have reduced access to preventive health services and face greater barriers to adopting healthy lifestyles, indirectly contributing to an unfavorable lipid profile [15,16].

Lifestyle factors exert a profound influence on atherogenic risk. Diets rich in saturated fats, added sugars, and ultra-processed foods are linked to adverse changes in lipid fractions, while adherence to cardioprotective dietary patterns, such as the Mediterranean diet, is consistently associated with lower lipid ratios and reduced AD prevalence [17,18]. Regular physical activity, particularly aerobic exercise, improves insulin sensitivity, increases HDL-c, and reduces triglycerides and other atherogenic particles [19]. In contrast, physical inactivity and chronic smoking are strongly related to higher TG/HDL-c values and increased AD prevalence [20,21].

In recent years, psychosocial factors such as social isolation and perceived loneliness have gained attention as potential contributors to cardiovascular risk [22]. Social isolation may influence atherogenic risk through behavioral pathways—such as reduced engagement in healthy activities—and biological mechanisms, including heightened hypothalamic–pituitary–adrenal axis activity and low-grade systemic inflammation [23,24]. Incorporating these psychosocial dimensions into the study of AD may offer a more comprehensive understanding of its etiology and potential preventive strategies.

Despite the substantial body of research addressing traditional cardiovascular risk factors, fewer studies have examined the combined influence of sociodemographic characteristics, lifestyle behaviors, and social isolation on atherogenic lipid ratios in large occupational cohorts. The working population represents a particularly relevant context, as it is shaped by occupational exposures, socioeconomic constraints, and lifestyle patterns that may jointly influence cardiovascular risk [25]. Furthermore, workplace-based health surveillance offers unique opportunities for early detection of adverse lipid profiles and the implementation of targeted preventive interventions.

In this context, the current research seeks to examine the relationships between sociodemographic characteristics, lifestyle behaviors, and social isolation with three well-established lipid ratios (TC/HDL-C, LDL-C/HDL-C, and TG/HDL-C), as well as with the occurrence of atherogenic dyslipidemia, in a large cohort of Spanish employees. Uncovering factors associated with higher atherogenic risk may provide valuable insights for the development of targeted and context-sensitive preventive strategies, thereby helping to reduce the cardiovascular disease burden within the workplace environment.

## 2. Methods

### 2.1. Study Design and Population

**Figure 1:**
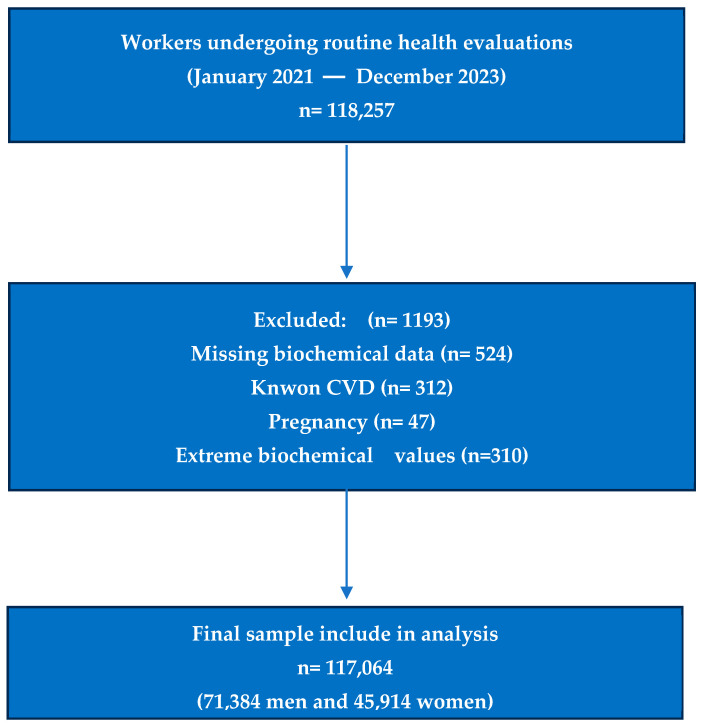
Flowchart of study selection.

This cross-sectional analysis was based on a study population consisted of Spanish workers attending routine occupational health check-ups between January 2021 and December 2024. Ensuring standardized procedures for data collection and comparability across participants. The study population derived from the same multicenter occupational health surveillance program described previously for cardiometabolic re-search in Spain, which collects sociodemographic, clinical, anthropometric, biochemical, and lifestyle data using harmonized protocols. Participants with incomplete data on lipid profiles or key sociodemographic variables were excluded. Although most participants were free of cardiovascular disease, a proportion were receiving medications that could influence lipid metabolism, including statins, antihypertensives, or antidiabetic agents. The use of lipid-lowering treatment (mainly statins) was recorded and included as a covariate in multivariable analyses to minimize confounding effects [26,27] (Figure 1).

Eligible participants were men and women aged 18–69 years with complete data on fasting lipid profile, anthropometric measurements, and lifestyle variables. Exclusion criteria included pregnancy, missing biochemical data, known cardiovascular disease, or extreme biochemical values suggestive of acute illness. After applying exclusion criteria, the final analytic sample comprised 117,064 workers from diverse economic sectors, predominantly commerce, industry, and services (Table S1).

### 2.2. Anthropometric and Biochemical Measurements

Height and weight were measured using a SECA 700 stadiometer and balance scale (SECA GmbH, Hamburg, Germany), with participants in light clothing and without shoes. Body mass index (BMI) was calculated as weight (kg) divided by height squared (m^2^). Waist and hip circumferences were measured following World Health Organization (WHO) recommendations [28] with a non-elastic SECA tape (SECA, Chino, CA, USA), and waist-to-hip ratio (WHR) was derived.

Blood pressure was measured in a seated position after at least 5 min of rest, using an OMRON M6 automated sphygmomanometer (Omron Healthcare, Kyoto, Japan), with the average of two consecutive measurements recorded.

Biochemical parameters, including triglycerides, total cholesterol, HDL-c, and LDL-c, were determined from venous blood samples after 12 h of fasting, using enzymatic colorimetric assays on a Roche Cobas 8000 modular analyzer (Roche Diagnostics, Basel, Switzerland),certified by external quality programs [29,30].

### 2.3. Atherogenic Risk Indices

Three validated lipid ratios were calculated to assess atherogenic risk:

- TC/HDL-c ratio: total cholesterol divided by HDL-c concentration [31]
- LDL-c/HDL-c ratio: LDL-c divided by HDL-c concentration [32]
- TG/HDL-c ratio: triglycerides divided by HDL-c concentration [33]

For the purposes of this study, atherogenic dyslipidemia (AD) was operationally defined by the coexistence of hypertriglyceridemia (TG ≥ 150 mg/dL), reduced high-density lipoprotein cholesterol (HDL-C < 40 mg/dL in men and <50 mg/dL in women), and the presence of small, dense low-density lipoprotein (LDL-C) particles within reference limits. This definition reflects established clinical thresholds and has been widely applied in epidemiological research to characterize lipid abnormalities associated with increased cardiovascular risk [34].

The thresholds applied to classify the different atherogenic ratios were defined as follows. For the total cholesterol to HDL-C ratio (TC/HDL-C), values < 5 in men and <4.5 in women were categorized as low risk, 5–9 in men and 4.5–7 in women as moderate risk, and >9 in men and >7 in women as high risk. Regarding the LDL-C to HDL-C ratio (LDL-C/HDL-C), values < 3 indicated low risk, whereas values ≥ 3 were considered high risk. For the triglycerides to HDL-C ratio (TG/HDL-C), a cutoff point of ≥3 was established to denote high risk [35,36,37,38].

### 2.4. Sociodemographic, Lifestyle, and Social Isolation Variables

Sociodemographic variables comprised sex, age—stratified into four predefined groups (18–39, 40–49, 50–59, and 60–69 years)—and occupational social class. The latter was assigned using the Spanish National Classification of Occupations (CNO-11) and subsequently categorized in accordance with the framework proposed by the Spanish Society of Epidemiology, which is widely employed in population-based and occupational health research to ensure comparability across studies [39].

### 2.5. Lifestyle Factors Comprised

- Smoking status (current smoker, non-smoker) [40];
- Adherence to the Mediterranean dietary pattern was measured using the validated 14-item Mediterranean Diet Adherence Screener (MEDAS). This instrument, widely applied in both epidemiological and clinical research, captures the frequency of consumption of key components of the Mediterranean diet. In accordance with established criteria, a total score of ≥9 points was used to classify participants as having high adherence to this dietary model, a threshold consistently associated with beneficial cardiometabolic outcomes [41];
- Physical activity was assessed through the short form of the International Physical Activity Questionnaire (IPAQ), a tool validated for use in diverse populations and frequently employed in large-scale epidemiological studies. The questionnaire provides information on the frequency and duration of moderate- and vigorous-intensity activities as well as walking and sedentary behavior. For the purposes of this study, participants were dichotomized into two categories—active and inactive—according to established scoring protocols [42].

Social isolation was evaluated using the validated ENRICHD Social Support Instrument (ESSI), categorized as low vs. normal [43].

### 2.6. Statistical Analysis

Continuous variables are presented as mean ± standard deviation (SD) and categorical variables as frequencies and percentages. Sex-specific comparisons were conducted using Student’s *t* test for continuous variables and χ^2^ tests for categorical variables.

Mean lipid ratio values were compared across sociodemographic, lifestyle, and social isolation categories using analysis of variance (ANOVA) with post hoc Bonferroni correction. Logistic regression models estimated odds ratios (OR) and 95% confidence intervals (CI) for moderate-to-high lipid ratios and AD, adjusting for age, sex, social class, smoking, Mediterranean diet adherence, physical activity, and social isolation. Model fit was assessed using the Hosmer–Lemeshow test, and multicollinearity was evaluated via variance inflation factors (VIF).

All analyses were performed using SPSS Statistics version 29.0 (IBM Corp., Armonk, NY, USA). A *p* value < 0.05 was considered statistically significant.

## 3. Results

Table 1 provides a comprehensive baseline characterization of the cohort, stratified by sex. Men exhibited higher mean triglyceride concentrations (133.4 ± 92.1 mg/dL) compared with women (91.1 ± 48.4 mg/dL), whereas HDL-c values were significantly lower in men (49.5 ± 6.9 mg/dL) versus women (52.6 ± 7.4 mg/dL, *p* < 0.001). Similarly, the prevalence of atherogenic dyslipidemia was nearly fourfold higher among smokers (12.1% in men and 2.8% in women) compared with non-smokers (2.7% in men and 2.7% in women, *p* < 0.001). These data provide a clearer understanding of the magnitude of the associations described. These baseline differences are relevant for interpreting subsequent associations between atherogenic indices and covariates, as they highlight potential sex-specific pathways in lipid metabolism and cardio-vascular risk.

The data reveal clear gradients in atherogenic lipid ratios across age groups, with progressive increases in TC/HDL-c and LDL-c/HDL-c with advancing age for both sexes, while TG/HDL-c rises less steeply in women. Lifestyle-related disparities are evident: participants adhering to the Mediterranean diet or engaging in regular physical activity consistently showed markedly lower atherogenic ratios, underscoring the protective role of healthy behaviors. Smoking and low social isolation were associated with higher TG/HDL-c values, particularly in men, suggesting a potential clustering of adverse behavioral and psychosocial factors that amplify atherogenic risk (Table 2).

Table 3 is presented separately for men (upper section) and women (lower section) to highlight sex-specific prevalence patterns of moderate-to-high lipid ratios and atherogenic dyslipidemia across sociodemographic, lifestyle, and social isolation categories. Highlight the pronounced influence of age on atherogenic dyslipidemia (AD), with prevalence estimates nearly doubling from younger to older categories, thereby reinforcing the cumulative cardiometabolic burden associated with aging. Lifestyle determinants also exhibited robust associations: limited adherence to the Mediterranean dietary model and physical inactivity were systematically linked to elevated lipid ratios and increased prevalence of AD, consistent with the protective role of healthy dietary patterns and regular physical exercise described in the previous literature. Tobacco consumption emerged as a particularly relevant factor, as smokers demonstrated substantially higher rates of TG/HDL-C elevation and AD, with the magnitude of this association being more accentuated in men—an observation that aligns with evidence suggesting sex-specific vulnerabilities in lipid metabolism. Additionally, reduced social connectedness was correlated with adverse lipid profiles, indicating that social isolation may constitute an independent risk factor for atherogenic alterations. This relationship could be explained by behavioral mediators, such as poorer adherence to healthy lifestyles, or by stress-induced neuroendocrine mechanisms, both of which warrant further investigation. Collectively, these findings emphasize the importance of integrating sociodemographic, behavioral, and psychosocial dimensions when designing interventions aimed at reducing atherogenic risk in working populations.

Table 4 shows the results of adjusted multivariable logistic regression analyses. All models were simultaneously adjusted for age, sex, social class, smoking status, Mediterranean diet adherence, physical activity, and social isolation. This approach ensures that the observed associations represent the independent contribution of each factor, controlling for potential confounders.

Multivariable logistic regression confirms the independent contribution of several risk factors to elevated lipid ratios and AD. Age and male sex were strong predictors for TC/HDL-c and TG/HDL-c elevation, while lower social class was associated with higher odds for all atherogenic measures, highlighting the role of social determinants in cardiovascular risk. The protective effect of Mediterranean diet adherence and physical activity was robust across all models, with odds reductions exceeding 50% in some comparisons. Smoking emerged as a powerful risk factor for TG/HDL-c elevation and AD, with adjusted ORs > 3 in some cases. Low social isolation remained significantly associated with elevated ratios even after adjustment, supporting its inclusion as a relevant variable in cardiovascular risk assessments.

**Figure 2:**
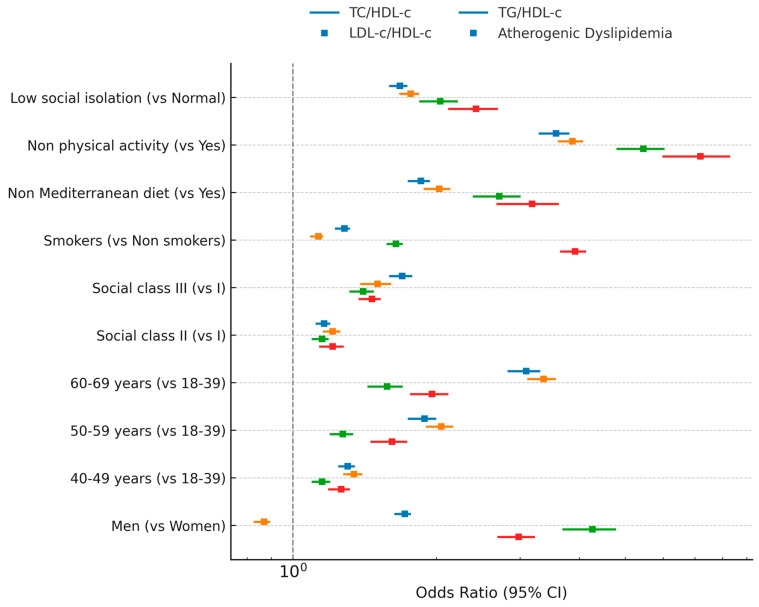
Forest plot of atherogenic risk indicators.

In the forest plot, we can see the ORs of the different atherogenic risk indicators (Figure 2).

## 4. Discussion

### 4.1. Main Findings

In this large occupational cohort of Spanish workers, we observed significant associations between sociodemographic characteristics, lifestyle factors, and social isolation with atherogenic lipid ratios (TC/HDL-c, LDL-c/HDL-c, TG/HDL-c) and the prevalence of atherogenic dyslipidemia (AD). Age and male sex were consistently related to higher lipid ratios, while adherence to the Mediterranean diet and regular physical activity were strongly protective. Smoking and low social isolation were associated with markedly worse atherogenic profiles. These associations remained robust after multivariable adjustment, underscoring the independent contributions of behavioral and psychosocial determinants to atherogenic risk in the working population.

### 4.2. Comparison with Previous Studies

Our results are in line with prior research showing that AD is more prevalent in men and increases with age [44]. The observed sex differences may reflect a combination of hormonal, genetic, and behavioral factors, as well as differential exposure to lifestyle risks [45]. The protective effect of Mediterranean diet adherence is consistent with studies demonstrating improvements in HDL-c concentrations, reductions in triglycerides, and favorable changes in lipoprotein particle size [46].

The strong association between physical inactivity and elevated atherogenic ratios parallels evidence from population-based cohorts where low cardiorespiratory fitness and sedentary behavior were linked to unfavorable lipid profiles [47]. Similarly, smoking has been shown to reduce HDL-c levels, increase triglycerides, and alter LDL particle composition, thereby exacerbating atherogenic risk [48].

Our findings regarding social isolation extend existing literature, as few occupational studies have simultaneously addressed psychosocial factors alongside biochemical markers of atherogenic risk [49]. The positive association between low social integration and adverse lipid ratios aligns with reports linking social isolation to increased cardiovascular morbidity and mortality [50].

### 4.3. Potential Mechanisms

Several biological pathways may underlie the associations observed. Elevated TC/HDL-c, LDL-c/HDL-c, and TG/HDL-c ratios reflect an imbalance between pro- and anti-atherogenic lipoproteins, which promotes endothelial dysfunction, vascular inflammation, and plaque formation [51]. Lifestyle factors such as poor diet quality and physical inactivity may exacerbate this imbalance by increasing hepatic VLDL production, reducing HDL synthesis, and impairing lipid clearance [52].

Smoking contributes to oxidative stress, inflammation, and lipoprotein oxidation, while also lowering HDL-c levels [53,54]. Chronic social isolation may influence lipid metabolism through behavioral pathways (e.g., unhealthy dietary habits, reduced physical activity) and through physiological stress responses that upregulate inflammatory and neuroendocrine activity, further promoting an atherogenic milieu [55,56].

### 4.4. Strengths and Limitations

The strengths of our study include its large sample size, standardized assessment of anthropometric and biochemical parameters, and comprehensive evaluation of lifestyle and psychosocial factors. The use of validated lipid ratios and a well-accepted definition of AD enhances the clinical relevance of our findings. Moreover, the occupational setting allowed the assessment of a broad range of socioeconomic groups, improving the generalizability to the working population.

However, several limitations must be considered. The cross-sectional design precludes causal inference. Residual confounding cannot be excluded, despite adjustment for multiple covariates. The definition of AD was based on lipid profiles rather than direct LDL particle measurement, which may lead to some misclassification. Finally, lifestyle and social isolation data were self-reported, introducing potential recall bias.

### 4.5. Implications for Public Health and Future Research

These findings highlight the importance of integrating atherogenic lipid ratios and AD assessment into occupational health programs, alongside interventions promoting healthy diet, regular physical activity, smoking cessation, and social engagement. Given the significant impact of AD on cardiovascular risk, workplace-based strategies targeting modifiable behaviors could substantially reduce disease burden.

Future research should employ longitudinal designs to confirm these associations and explore whether changes in lifestyle and social integration can favorably modify atherogenic risk profiles over time. Moreover, studies incorporating direct measurements of lipoprotein subfractions and inflammatory markers could provide a deeper understanding of the mechanisms linking psychosocial and lifestyle factors with lipid metabolism.

## 5. Conclusions

In this large cohort of Spanish workers, atherogenic lipid ratios and atherogenic dyslipidemia were strongly influenced by sociodemographic characteristics, lifestyle behaviors, and social isolation. Age, male sex, smoking, and lower social engagement were associated with a markedly less favorable lipid profile, whereas adherence to the Mediterranean diet and regular physical activity were linked to protective effects. These findings underscore the importance of incorporating atherogenic risk assessment into occupational health evaluations and designing workplace interventions that target modifiable risk factors.

Considering the well-documented contribution of atherogenic dyslipidemia to the development of cardiovascular disease, its early detection and management in the working-age population may yield significant benefits for long-term health and disease prevention. Proactive strategies targeting this group could help attenuate the progression of cardiometabolic disorders before clinical complications emerge. Nevertheless, longitudinal investigations are needed to establish causal pathways and to assess the efficacy of comprehensive interventions—encompassing lifestyle modification and psychosocial support—in improving lipid ratios and ultimately lowering cardiovascular risk.

## Figures and Tables

**Table 1:** Anthropometric, clinical, and lifestyle characteristics of the study population by sex.

	Men *n* = 71,384	Women *n* = 45,914	
Variables	Mean (SD)	Mean (SD)	*p*-Value
Age (years)	45.5 (7.4)	45.2 (7.2)	<0.001
Height (cm)	173.1 (7.0)	160.2 (6.5)	<0.001
Weight (kg)	82.2 (13.5)	66.0 (12.9)	<0.001
Waist (cm)	88.5 (9.2)	74.4 (7.9)	<0.001
Hip (cm)	100.5 (8.3)	97.7 (8.7)	<0.001
SBP (mm Hg)	126.4 (15.7)	116.7 (15.4)	<0.001
DBP (mm Hg)	77.4 (10.6)	71.3 (10.5)	<0.001
Cholesterol (mg/dL)	205.0 (37.3)	201.4 (36.0)	<0.001
HDL-c (mg/dL)	49.5 (6.9)	52.6 (7.4)	<0.001
LDL-c (mg/dL)	129.1 (36.6)	130.7 (36.4)	<0.001
Triglycerides (mg/dL)	133.4 (92.1)	91.1 (48.4)	<0.001
Glucose (mg/dL)	90.0 (13.2)	85.8 (11.8)	<0.001
Variables	*n* (%)	*n* (%)	*p*-value
18–39 years	18,418 (25.8)	12,214 (26.6)	<0.001
40–49 years	32,098 (45.0)	20,934 (45.6)	
50–59 years	17,350 (24.5)	11,094 (24.2)	
60–69 years	3338 (4.7)	1672 (3.6)	
Social class I	4002 (5.6)	2980 (6.5)	<0.001
Social class II	12,978 (18.2)	13,856 (30.2)	
Social class III	54,404 (76.2)	29,078 (63.3)	
Smokers	24,426 (34.2)	14,132 (30.8)	<0.001
Yes Mediterranean diet	22,858 (32.0)	20,536 (44.7)	<0.001
Yes physical activity	26,010 (36.4)	20,478 (45.2)	<0.001
Social isolation low	27,376 (38.4)	4198 (9.1)	<0.001
Social isolation normal	44,008 (61.6)	41,716 (90.9)	

SBP: systolic blood pressure. DBP: diastolic blood pressure. HDL: high-density lipoprotein. LDL: low-density lipoprotein. SD: standard deviation.

**Table 2:** Mean values of atherogenic lipid ratios (TC/HDL-c, LDL-c/HDL-c, TG/HDL-c) by sociodemographic, lifestyle, and social isolation variables, stratified by sex.

		TC/HDL-c		LDL-c/HDL-c		TG/HDL-c	
Men	*n*	Mean (SD)	*p*-Value	Mean (SD)	*p*-Value	Mean (SD)	*p*-Value
18–39 years	18,418	3.9 (1.0)	<0.001	2.4 (0.9)	<0.001	2.5 (2.1)	<0.001
40–49 years	32,098	4.2 (1.1)		2.7 (0.9)		2.9 (2.5)	
50–59 years	17,350	4.5 (1.2)		2.9 (1.0)		3.0 (2.1)	
60–69 years	3338	4.6 (1.2)		3.0 (1.0)		3.1 (2.4)	
Social class I	4002	4.1 (1.0)	<0.001	2.6 (0.9)	<0.001	2.6 (2.4)	<0.001
Social class II	12,978	4.2 (1.1)		2.7 (0.9)		2.8 (2.3)	
Social class III	54,404	4.3 (1.2)		2.7 (1.0)		2.9 (2.4)	
Smokers	24,426	4.2 (1.0)	<0.001	2.7 (0.9)	<0.001	2.6 (1.9)	<0.001
Non-smokers	46,778	4.4 (1.3)		2.8 (1.1)		3.3 (3.0)	
Yes MD	22,858	3.6 (0.6)		2.3 (0.6)		1.7 (0.7)	
Non-MD	48,346	4.6 (1.2)		2.9 (1.0)		3.4 (2.7)	
Yes PhA	26,010	3.6 (0.6)	<0.001	2.3 (0.6)	<0.001	1.7 (0.6)	<0.001
Non-PhA	45,194	4.6 (1.2)		2.9 (1.1)		3.5 (2.7)	
Social isolation low	27,376	4.5 (1.2)	<0.001	2.8 (1.0)	<0.001	3.5 (2.9)	<0.001
Social isolation normal	44,008	4.1 (1.1)		2.6 (0.9)		2.4 (1.8)	
women	*n*	Mean (SD)	*p*-value	Mean (SD)	*p*-value	Mean (SD)	*p*-value
18–39 years	12,214	3.5 (0.9)	<0.001	2.2 (0.8)	<0.001	1.6 (0.9)	<0.001
40–49 years	20,934	3.9 (1.0)		2.5 (0.9)		1.8 (1.0)	
50–59 years	11,094	4.4 (1.1)		3.0 (1.0)		2.0 (1.2)	
60–69 years	1672	4.5 (1.0)		3.1 (0.9)		2.2 (1.1)	
Social class I	2980	3.7 (1.0)	<0.001	2.4 (0.9)	<0.001	1.6 (0.9)	<0.001
Social class II	13,856	3.9 (1.0)		2.5 (0.9)		1.7 (1.0)	
Social class III	29,078	4.0 (1.1)		2.6 (1.0)		1.9 (1.1)	
Smokers	14,132	4.0 (1.1)	<0.001	2.6 (1.0)	<0.001	1.7 (1.0)	<0.001
Non-smokers	31,781	3.9 (1.0)		2.5 (1.0)		1.9 (1.1)	
Yes MD	20,536	3.4 (0.7)		2.2 (0.7)		1.4 (0.5)	
Non-MD	25,377	4.3 (1.1)		2.9 (1.0)		2.1 (1.3)	
Yes PhA	20,478	3.4 (0.7)	<0.001	2.2 (0.6)	<0.001	1.3 (0.2)	<0.001
Non-PhA	25,155	4.4 (1.1)		2.9 (1.0)		2.2 (1.3)	
Social isolation low	4198	4.4 (1.1)	<0.001	2.9 (1.0)	<0.001	2.5 (1.5)	<0.001
Social isolation normal	41,716	3.9 (1.0)		2.6 (1.0)		1.7 (1.0)	

TC: total cholesterol. HDL-c: high-density lipoprotein-cholesterol. LDL-c: low-density lipoprotein- cholesterol. TG: triglycerides. MD: Mediterranean diet. PhA: physical activity. SD: standard deviation.

**Table 3:** Prevalence of moderate-to-high atherogenic ratios and atherogenic dyslipidemia by sociodemographic, lifestyle, and social isolation variables, stratified by sex.

		TC/HDL-c Moderate-High		LDL-c/HDL-c High		TG/HDL-c High		AD	
Men	*n*	%	*p*-Value	%	*p*-Value	%	*p*-Value	%	*p*-Value
18–39 years	18,418	10.9	<0.001	19.6	<0.001	22.3	<0.001	4.2	<0.001
40–49 years	32,098	18.6		30.8		30.0		5.8	
50–59 years	17,350	28.8		43.9		35.5		7.6	
60–69 years	3338	29.2		44.9		35.8		8.4	
Social class I	4002	18.3	<0.001	30.4	<0.001	23.5	<0.001	4.1	<0.001
Social class II	12,978	18.8		32.1		28.6		5.3	
Social class III	54,404	20.1		32.9		30.4		6.2	
Smokers	24,426	21.9	<0.001	33.2	<0.001	34.8	<0.001	12.1	<0.001
Non-smokers	46,778	18.5		31.7		27.1		2.7	
Yes MD	22,858	10.7		11.3		20.1		4.1	
Non-MD	48,346	22.5		41.5		42.0		10.8	
Yes PhA	26,010	8.8	<0.001	12.3	<0.001	16.3	<0.001	2.9	<0.001
Non-PhA	45,194	26.9		43.0		46.5		12.5	
Social isolation low	27,376	26.2	<0.001	37.6	<0.001	41.5	<0.001	9.5	<0.001
Social isolation normal	44,008	15.6		28.2		22.4		3.7	
women	*n*	%	*p*-value	%	*p*-value	%	*p*-value	%	*p*-value
18–39 years	12,214	14.1	<0.001	16.2	<0.001	5.3	<0.001	1.2	<0.001
40–49 years	20,934	23.9		26.3		8.3		2.2	
50–59 years	11,094	41.5		45.3		14.5		5.2	
60–69 years	1672	45.7		48.6		18.1		5.4	
Social class I	2980	20.2	<0.001	27.0	<0.001	6.4	<0.001	2.2	<0.001
Social class II	13,856	24.5		27.3		7.8		2.3	
Social class III	29,078	27.9		30.6		10.4		3.1	
Smokers	14,132	26.5	<0.001	29.5	<0.001	10.7	<0.001	2.8	<0.001
Non-smokers	31,781	26.1		27.9		8.7		2.7	
Yes MD	20,536	16.5		11.3		5.6		1.9	
Non-MD	25,377	32.6		43.3		14.2		4.0	
Yes PhA	20,478	12.0	<0.001	9.9	<0.001	4.4	<0.001	1.1	<0.001
Non-PhA	25,155	38.9		44.8		16.2		4.9	
Social isolation low	4198	40.4	<0.001	41.5	<0.001	24.0	<0.001	4.1	<0.001
Social isolation normal	41,716	24.9		27.7		8.9		2.5	

TC: total cholesterol. HDL:-c high-density lipoprotein-cholesterol. LDL-c: low-density lipoprotein- cholesterol. TG: triglycerides. AD: atherogenic dyslipidemia. MD: Mediterranean diet. PhA: physical activity. The upper part corresponds to male participants and the lower part to female participants, allowing a direct sex-stratified comparison.

**Table 4:** Adjusted odds ratios for moderate-to-high atherogenic ratios and atherogenic dyslipidemia by sociodemographic, lifestyle, and social isolation variables.

	TC/HDL-c Moderate-High		LDL-c/HDL-c		TG/HDL-c		AD	
	OR (95% CI)	*p*-Value	OR (95% CI)	*p*-Value	OR (95% CI)	*p*-Value	OR (95% CI)	*p*-Value
Women	1		1		1		1	
Men	1.70 (1.64–1.76)	<0.001	0.86 (0.83–0.89)	<0.001	4.22 (3.70–4.75)	<0.001	2.95 (2.70–3.21)	<0.001
18–39 years	1		1		1		1	
40–49 years	1.29 (1.25–1.34)	<0.001	1.33 (1.28–1.39)	<0.001	1.14 (1.10–1.19)	<0.001	1.25 (1.19–1.31)	<0.001
50–59 years	1.87 (1.75–1.99)	<0.001	2.03 (1.91–2.16)	<0.001	1.26 (1.20–1.33)	<0.001	1.60 (1.46–1.73)	<0.001
60–69 years	3.06 (2.84–3.29)	<0.001	3.33 (3.12–3.55)	<0.001	1.56 (1.44–1.69)	<0.001	1.94 (1.77–2.11)	<0.001
Social class I	1		1		1		1	
Social class II	1.15 (1.12–1.19)	<0.001	1.20 (1.16–1.25)	<0.001	1.14 (1.10–1.18)	<0.001	1.20 (1.14–1.27)	<0.001
Social class III	1.68 (1.60–1.77)	<0.001	1.49 (1.39–1.60)	<0.001	1.39 (1.32–1.47)	<0.001	1.45 (1.38–1.52)	<0.001
Non-smokers	1		1		1		1	
Smokers	1.27 (1.23–1.31)	<0.001	1.12 (1.09–1.15)	<0.001	1.63 (1.58–1.69)	<0.001	3.88 (3.66–4.11)	<0.001
Yes Mediterranean diet	1		1		1		1	
Non-Mediterranean diet	1.84 (1.75–1.93)	<0.001	2.01 (1.89–2.13)	<0.001	2.69 (2.40–2.99)	<0.001	3.15 (2.69–3.60)	<0.001
Yes physical activity	1		1		1		1	
Non-physical activity	3.54 (3.30–3.79)	<0.001	3.83 (3.62–4.05)	<0.001	5.40 (4.81–6.01)	<0.001	7.12 (6.01–8.25)	<0.001
Social isolation normal	1		1		1		1	
Social isolation low	1.66 (1.60–1.73)	<0.001	1.75 (1.68–1.83)	<0.001	2.02 (1.85–2.21)	<0.001	2.40 (2.13–2.68)	<0.001

TC: total cholesterol. HDL-c: high-density lipoprotein-cholesterol. LDL-c: low-density lipoprotein- cholesterol. TG: triglycerides. AD: atherogenic dyslipidemia. OR: odds ratio. CI: confidence interval.

## Data Availability

Due to ethical and legal constraints, particularly the protection of sensitive personal health information, the datasets generated and analyzed in this study are not publicly available. Data may be obtained from the corresponding author upon reasonable request, in compliance with Spanish regulations and the European Union General Data Protection Regulation (GDPR).

## Supplementary Materials

The following supporting information can be downloaded at: https://www.mdpi.com/article/10.3390/jcm14197039/s1, Table S1: Baseline characteristics of the study population (*n* = 117,064).
