# Supplementary material for: Determinants of Atherogenic Dyslipidemia and Lipid Ratios: Associations with Sociodemographic Profile, Lifestyle, and Social Isolation in Spanish Workers
Source: J Clin Med. 2025 Oct 5;14(19):7039. doi: 10.3390/jcm14197039 (PMC12524693; doi:10.3390/jcm14197039)
Supplement: Supplementary file 1 [file jcm-14-07039-s001.zip › jcm-3871131-supplementary.pdf]

## Supplementary Table S1. Baseline characteristics of the study population (n = 117,064)

| Variable | Total (n = 117,064) | Men (n = 71,384) | Women (n = 45,680) |
|---|---|---|---|
| Age, years (mean ± SD) | 45.4 ± 7.3 | 45.5 ± 7.4 | 45.2 ± 7.2 |
| Age groups, n (%) | | | |
| 18–39 years | 30,632 (26.2) | 18,418 (25.8) | 12,214 (26.6) |
| 40–49 years | 53,032 (45.3) | 32,098 (45.0) | 20,934 (45.6) |
| 50–59 years | 28,444 (24.3) | 17,350 (24.5) | 11,094 (24.2) |
| 60–69 years | 5,010 (4.3) | 3,338 (4.7) | 1,672 (3.6) |
| BMI, $kg/m^2$ (mean ± SD) | 25.7 ± 4.5 | 26.8 ± 4.2 | 24.0 ± 4.5 |
| Smoking status, n (%) | | | |
| Current smokers | 38,558 (32.9) | 24,426 (34.2) | 14,132 (30.8) |
| Non-smokers | 78,506 (67.1) | 46,958 (65.8) | 31,548 (69.2) |
| Mediterranean diet adherence, n (%) | | | |
| High (≥9 points MEDAS) | 43,394 (37.1) | 22,858 (32.0) | 20,536 (44.7) |
| Low (<9 points MEDAS) | 73,670 (62.9) | 48,526 (68.0) | 25,144 (55.3) |
| Physical activity (IPAQ), n (%) | | | |
| Active | 46,488 (39.7) | 26,010 (36.4) | 20,478 (45.2) |
| Inactive | 70,576 (60.3) | 45,374 (63.6) | 25,202 (54.8) |
| Social class, n (%) | | | |
| Class I | 6,982 (6.0) | 4,002 (5.6) | 2,980 (6.5) |
| Class II | 26,834 (22.9) | 12,978 (18.2) | 13,856 (30.2) |
| Class III | 83,248 (71.1) | 54,404 (76.2) | 28,844 (63.3) |
| Social isolation (ESSI), n (%) | | | |
| Low | 31,574 (27.0) | 27,376 (38.4) | 4,198 (9.1) |
| Normal | 85,490 (73.0) | 44,008 (61.6) | 41,482 (90.9) |
| Medication use, n (%) | | | |
| Statins (lipid-lowering agents) | 8,312 (7.1) | 5,640 (7.9) | 2,672 (5.9) |
| Antihypertensives | 10,454 (8.9) | 6,822 (9.6) | 3,632 (7.9) |
| Antidiabetic drugs | 5,398 (4.6) | 3,684 (5.2) | 1,714 (3.8) |
